# A *retro*-*inverso* cell-penetrating peptide for siRNA delivery

**DOI:** 10.1186/s12951-017-0269-2

**Published:** 2017-04-28

**Authors:** Anaïs Vaissière, Gudrun Aldrian, Karidia Konate, Mattias F. Lindberg, Carole Jourdan, Anthony Telmar, Quentin Seisel, Frédéric Fernandez, Véronique Viguier, Coralie Genevois, Franck Couillaud, Prisca Boisguerin, Sébastien Deshayes

**Affiliations:** 1 Centre de Recherche de Biologie cellulaire de Montpellier, UMR 5237 CNRS, 1919 Route de Mende, 34293 Montpellier, France; 2 Sys2Diag, UMR 9005-CNRS/ALCEDIAG, 1682 Rue de la Valsiere, 34184 Montpellier, France; 3 Microscopie Électronique et Analytique, Université de Montpellier, Place Eugène Bataillon, 34095 Montpellier, France; 4 EA 7435 IMOTION (Imagerie moléculaire et thérapies innovantes en oncologie), Université de Bordeaux, 146 rue Leo Saignat, 33076 Bordeaux, France

**Keywords:** Enantiomer, d-Amino acids, Retro-inverso, siRNA delivery, Cell penetrating peptides, Nanoparticle, Gene knock-down, Cancer

## Abstract

**Background:**

Small interfering RNAs (siRNAs) are powerful tools to control gene expression. However, due to their poor cellular permeability and stability, their therapeutic development requires a specific delivery system. Among them, cell-penetrating peptides (CPP) have been shown to transfer efficiently siRNA inside the cells. Recently we developed amphipathic peptides able to self-assemble with siRNAs as peptide-based nanoparticles and to transfect them into cells. However, despite the great potential of these drug delivery systems, most of them display a low resistance to proteases.

**Results:**

Here, we report the development and characterization of a new CPP named RICK corresponding to the *retro*-*inverso* form of the CADY-K peptide. We show that RICK conserves the main biophysical features of its L-parental homologue and keeps the ability to associate with siRNA in stable peptide-based nanoparticles. Moreover the RICK:siRNA self-assembly prevents siRNA degradation and induces inhibition of gene expression.

**Conclusions:**

This new approach consists in a promising strategy for future in vivo application, especially for targeted anticancer treatment (e.g. knock-down of cell cycle proteins).

**Graphical abstract:**
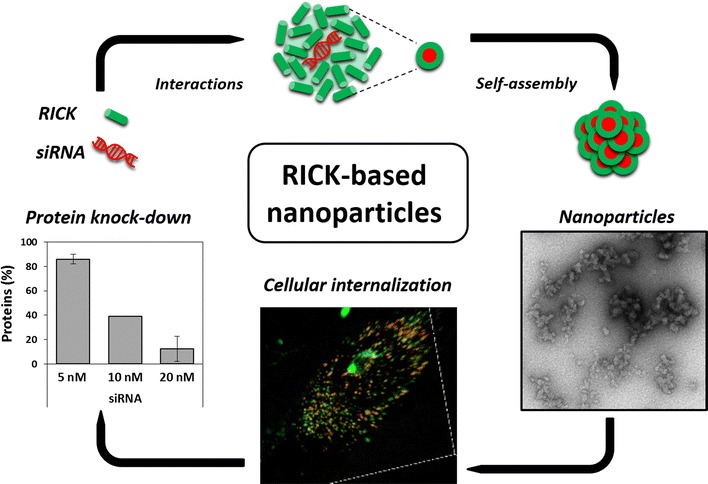
RICK-based nanoparticles: RICK peptides and siRNA self-assemble in peptide-based nanoparticles to penetrate into the cells and to induce target protein knock-down.

**Electronic supplementary material:**

The online version of this article (doi:10.1186/s12951-017-0269-2) contains supplementary material, which is available to authorized users.

## Background

Small interfering RNAs (siRNAs) have been widely considered as powerful tools to specifically control protein activation and/or gene expression post-transcriptionally [1], particularly for complex genotypic alterations occurring in cancer [2]. siRNAs can enter the RNA-induced silencing complex (RISC), which induces enzyme-catalyzed degradation of their complementary messenger RNAs (mRNAs) in cells, thus disrupting specific molecular pathways in various diseases [3]. The design versatility and the highly specific nature of these oligonucleotides highlight their potential as drugs of the future. However, biological barriers remain the main obstacle for the siRNAs delivery due to their large molecular weight (∼14 kDa) and their highly anionic (∼40 negative charges) character [4, 5].

Currently, most delivery strategies including lipids, polycationic polymers, nanoparticles and peptide-based formulations are based on formulations protecting siRNAs from degradation by hydrolytic enzymes and at the same time mediate their cellular delivery. Several technologies have been proposed to tackle these problems [6–8]. Cell penetrating peptides (CPPs) are one of the most promising non‐viral strategies to improve intracellular routing of large molecules including siRNA [6, 9, 10]. CPPs are usually short (up to 30 amino acids) peptides that originate from a wide variety of sources (e.g., humans, mice, viruses or purely synthetic) [6]. Based on their structural characteristics, CPPs can be divided into two classes: arginine-rich CPPs and amphipathic CPPs [11].

Amphipathic CPPs contain both hydrophilic and hydrophobic domains necessary for cellular internalization and interaction with the cargo. In primary amphipathic CPPs, these domains are distributed according to their position along the peptide chain as shown for pVEC [12], MPG and Pep-1 [13]. Secondary amphipathic CPPs are another large class of peptides in which the separation between hydrophilic and hydrophobic domains occurs due to the secondary structure formation, such as α helices or β sheets [13]. Many of the most commonly used CPPs are members of this class such as penetratin [14], transportan [15], CADY [16] or C6M1 [17].

Strategies based on the conjugation of siRNA moieties to the CPP lacked efficacy and activated innate immunity in vivo [18]. Furthermore, the incorporation of the siRNA guide strand into the deep-pocket of RISC, essential for the silencing mechanism, is inhibited by covalent linkage between the CPP and the siRNA [4]. Therefore, non-covalent approaches based on electrostatic and hydrophobic interactions between the CPP and the cargo resulting in the nanoparticle formation are currently in the focus of drug delivery strategies [17, 19–22].

Previous investigations revealed that the conformational state of non-covalent CPPs plays an important role in the interaction with the cargo as well as in the self-assembly process leading to efficient peptide-based nanoparticles (PBN) [23]. In addition, the secondary structure of CPPs seems to control membrane interactions, peptide and siRNA entry and to influence the final cellular internalization route [24]. In this context, our recent comparative study revealed that siRNA-loaded nanoparticles formed by the CADY-K CPP (derived from CADY [16]) displayed a twofold higher luciferase knock-down efficiency than the parental peptide or other analogues [25]. CADY-K is thus an ideal candidate for further application especially with regards to ex vivo or in vivo siRNA delivery.

However, the in vivo development of CPPs could be compromised by degradation phenomena resulting from extracellular and/or intracellular proteases, probably partly explaining the low success of CPP application in clinical trials. Therefore, we decided to further improve our applied strategy by using nanoparticles formulated with a *retro*-*inverso* CPP. In 1995, Goodman and Chorev first evaluated the advantages of *retro*-*inverso* peptides—peptides consisting in d-amino acids in the reverse sequence of the naturally occurring l-isoforms [26]. Subsequently, *retro*-*inverso* transformation has commonly been employed as a strategy for the synthesis of proteolytically stable peptide analogues while maintaining the structural features [27, 28]. Herein, we adopted a similar strategy to characterize the new CPP RICK (***R*** *etro*-***I*** *nverso* ***C*** *ADY*-***K***). Bearing a high degree of the topochemical equivalence of l-isomer CPP, the *retro*-*inverso* version would exhibit similar bioactivity as the corresponding l-peptide with the advantage of a reduced endogenous protease degradation.

Here, we present a more in-depth analysis of the structural conformation of the RICK CPP and of the nanoparticle formulation/characterization in the presence of siRNA. To better understand the structural feature and the new highly potent peptide-based nanoparticle (PBN) for siRNA delivery, homologues were also analyzed such as the l-isoform (CADY-K) and the d-isoform (d-cady-k). siRNA-loaded RICK nanoparticles were evaluated in vitro in terms of stability and protease-resistance as well as in vitro by evaluating the gene knock-down (luciferase and cyclin B1) in U87 human glioblastoma cells. Overall, our results provide a comprehensive molecular basis of siRNA-loaded RICK nanoparticles for the further development of PBNs based on *retro*-*inverso* CPPs.

## Methods

### Materials

Dioleylphosphatidylglycerol (DOPG), dioleylphosphatidylcholine (DOPC) and 1-palmitoyl,2-oleoylphosphatidylcholine (POPC) phospholipids, cholesterol (Chol), sphingomyelin (SM) and labelled phosphocholine (TopFluorPC) [29] were purchased from Avanti Polar Lipids. RICK, CADY-K, and d-cady-k were purchased from LifeTein (sequences in Table 1). Atto633-labeling of the RICK peptide was performed as described in Additional file 1. Unlabeled and Cy3-labeled siRNA were obtained from Eurogentec and siRNA-Cy3b (labelled on 3′-end) from BioSynthesis. The different sequences are for anti-firefly luciferase (siFLuc): 5′-CUU-ACG-CUG-AGU-ACU-UCG-AdTdT (sense strand) and a scrambled version of the anti-luciferase (siSCR): 5′-CAU-CAU-CCC-UGC-CUC-UAC-UdTdT-3′ (sense strand) used as control. The sequence for the siRNA anti-cyclinB1 (siCycB1) is 5′-GAA-AUG-UAC-CCU-CCA-GAA-AdTdT-3′ (sense strand). The *siRNA stock solutions* were prepared in RNase-free water. *Peptide stock solutions* as well as CPP:siRNA complexes were prepared as published recently [25]. *Large Unilamellar Vesicles (LUVs)* were prepared by the extrusion method from a lipid mixture of DOPC/SM/Chol (2:2:1) as previously reported [25]. *Giant Unilamellar Vesicles (GUVs)* were formed by using the hydration method [30] with several modification as mentioned in Additional file 1.

**Table 1:** RICK, CADY-K and d-cady-k characterization by DLS

Peptides			Mean size (nm) at 0 h			Mean size (nm) at 72 h		
ID	Sequences	Properties	I (%)	Nb (%)	PdI	I (%)	Nb (%)	PdI
RICK	kwllrwlsrllrwlarwlg	retro-inverso	92 ± 15	32 ± 4	0.24 ± 0.01	91 ± 14	37 ± 9	0.25 ± 0.02
CADY-K	GLWRALWRLLRSLWRLLWK	l-AA	116 ± 22	36 ± 3	0.30 ± 0.05	124 ± 26	28 ± 1	0.33 ± 0.07
d-Cady-k	glwralwrllrslwrllwk	d-aa	90 ± 16	28 ± 7	0.30 ± 0.02	82 ± 3	32 ± 2	0.29 ± 0.02

All CPP:siRNA complexes were formed at R = 20 with using a siRNA concentration of 500 nM in an aqueous solution of 5% glucose for mean size acquisition

### Structural evaluation

#### Circular dichroism (CD) measurements

CD spectra were recorded on a Jasco 810 (Japan) dichrograph in quartz suprasil cells (Hellma) with an optical path of 1 mm for peptide in solution or in the presence of liposomes vesicles. Same concentrations of peptide (40 µM) were used for each condition. Spectra were obtained from 3 accumulations between 190 and 260 nm with a data pitch of 0.5 nm, a bandwidth of 1 nm and a standard sensitivity.

#### Fluorescence spectrometry

Fluorescence experiments were performed on a PTI spectrofluorimeter at 25 °C in 5% glucose. Intrinsic Tryptophan fluorescence of RICK, CADY-K and d-cady-k at 5 µM was excited at 290 nm and emission spectrum was recorded between 320 and 390 nm, with a spectral band-pass of 2 and 6 nm for excitation and emission, respectively. Effect of siRNA was investigated through addition of siRNA to peptide solution in order to form complexes at a CPP:siRNA molar ratio of R = 20. All spectra are normalized to maximum of fluorescence of free peptides and plotted in relative fluorescence (%).

### Nanoparticle formation

#### Agarose gel shift assay

CPPs:siRNA complexes were formed at different ratio in 5% glucose and pre-incubated for 30 min at room temperature. Each sample was analyzed by agarose gel (1% w/v) electrophoresis stained with GelRed (Interchim) for UV detection as descripted previously [25].

#### Dynamic light scattering (DLS) and zeta potential (ZP)

CPPs:siRNA nanoparticles were evaluated with a Zetasizer NanoZS (Malvern) in terms of mean size (Z-average) of the particle distribution and of homogeneity (PdI). Zeta potential was determined in 5% glucose supplemented with 5 mM NaCl, OptiMEM or DMEM. All results were obtained from three independent measurements (three runs for each measurement at 25 °C).

#### Environmental scanning electron microscopy (ESEM) and transmission electron microscopy (TEM)

For environmental scanning electron microscopy experiments, RICK:siRNA complexes were especially formed in ultra-pure water in order to avoid interference due to 5% glucose. Complexes were prepared at a CPP:siRNA molar ratio of R = 20 and for a final peptide concentration of 40 µM. Drops of 10 µl were spotted onto a copper support. Particles were examined using an FEI Quanta 200 FEG Scanning Electron Microscope operated at 10.00 kV, with a pressure of 400 Pa and a magnification of 15,000 × (det Gaseous Scanning Electron Detector, Working Distance = 9.4 mm). For transmission electron microscopy (TEM), a drop of 5 µl of suspension is deposited on a carbon coated 300 mesh grid for 1 min, blotted dry by touching filter paper and then placed on a 2% uranyl acetate solution drop. After 1 min the excess stain is removed by touching the edge to a filter paper, the grid is dried at room temperature for few minutes and examined using a Jeol 1200EX2 Transmission Electron Microscope operating at 100 kV accelerating voltage. Data were collected with a SIS Olympus Quemesa CCD camera.

### Evaluation of the CPP stability

#### HPLC analysis

100 µl of each peptide (12 µM in water) were incubated with 20 µl of porcine trypsin (105 µM) at 37 °C. Evolution of the peptide in the presence of the protease was followed by RP-HPLC (Waters) at 220 nm on a C8 Aquapore RP-300 7 µ, 100 × 2.1 mm column (Perkin Elmer) at different time points. The mobile phase consisted of: A: demineralized water (Milli-Q quality; Millipore) and B: acetonitrile (HPLC gradient, Carlo Erba, Peypin), containing 0.1 and 0.08% trifluoroacetic acid (Sigma Aldrich), respectively. The elution profile was: from 0 to 60% B in 55 min with a flow rate of 1 ml/min. Injection volume was 20 µl.

#### Fluorescence spectrometry

CPP:siRNA-Cy3b nanoparticles (R = 20, with 20 nM of siRNA) formulated in 100 mM Tris–HCl (pH 7.5) were incubated on a non-binding black 96-well plate (Greiner Bio-One) with different volumes of 0.05% trypsin (Life Technologies). After 24 h incubation the fluorescence was recorded (Ex = 544 nm and Em = 590 nm).

#### siRNA extraction

CPP:siRNA-Cy3 nanoparticles (R = 20, with 200 pmol of siRNA) were incubated with fetal bovine serum (FBS) (PAA) 24 h at 37 °C. Thereafter the siRNA-Cy3 was extracted using the mirPremier™ microRNA isolation kit (Sigma-Aldrich) by following the instruction of the manufacturer. The siRNA-Cy3 was isolated on a denaturing urea (8 M) acrylamide gel (15%) and visualized using the pre-defined Cy3 filter on the Amersham Imager 600.

### Cellular activity evaluation of CPP:siRNA nanoparticles

#### Culture conditions

The efficiencies of siRNA-mediated gene silencing were investigated in U87 cell lines (U87 MG, human glioblastoma) stably transfected with firefly and *Renilla* luciferase (FLuc-RLuc) encoding plasmid (details of cell line generation are given in the Additional file 1). Cells were grown in a complete medium: DMEM (+GlutaMAX™ supplement, Life Technologies), with 100 units/ml penicillin (Life Technologies), 100 mg/ml streptomycin (Life Technologies), 10% heat-inactivated fetal bovine serum (FBS) (PAA), non-essential amino acids NEAA 1X (LifeTechnologies) and selection antibiotics hygromycine B (Invitrogen) (50 µg/ml) and Blasticidine (Gibco) (2 µg/ml). All the cells were maintained in a humidified incubator with 5% CO_2_ at 37 °C.

#### Transfection experiments

For Luciferase assay, 5000 cells were seeded 24 h before experiment into 96-well. The next day, nanoparticles were formed by mixing siRNA and CPPs (equal volumes, “siRNA on CPPs”) in 5% glucose water, followed by an incubation of 30 min at 37 °C. In the meantime, the growth medium covering the cells was replaced by 70 µl of fresh pre-warmed serum-free DMEM. 30 µl of the nanoparticle solutions were added directly to the cells and after 1 h 15 of incubation, 100 μl DMEM + 20% FBS was added to each well without withdrawing the transfection reagents, and cells were then incubated for another 36 h. The experimental procedure was designed to test CPP:siRNA nanoparticles at a peptide:siRNA molar ratio of R = 20, containing siRNA concentrations of 5, 10 and 20 nM in the final volume of 200 µl. For trypsin pre-incubation assay, CADY-K:siFLuc and RICK:siFLuc nanoparticles were incubated in the presence of trypsin (5 µg/ml and 10 µg/ml for nanoparticles loaded with 10 nM and 20 nM siRNA, R = 20) for 24 h at 37 °C. Before adding the nanoparticles solution to the cells, a trypsin inhibitor was added. Cells were incubated for 1.5 h with the nanoparticles in serum-free DMEM. After the addition of DMEM supplemented with 20% FBS (final FBS concentration = 10%), cells were further incubated for 48 h and finally lysed for the luciferase detection.

For Western blot assay, 75,000 cells were seeded 24 h before experiment into 24-well plates. For standard incubation, the cells were incubated with 175 µl of fresh pre-warmed serum-free DMEM + 75 µl of the nanoparticle solutions. After 1.5 h of incubation, 250 μl DMEM + 20% FBS was added to each well without withdrawing the transfection reagents, and cells were then incubated for another 24 h. The experimental procedure was designed to test CPP:siRNA nanoparticles at a peptide:siRNA molar ratio of R = 20, containing siRNA concentrations of 5, 10 and 20 nM in the final volume of 500 µl. For transfection in the presence of serum, cells were incubated with nanoparticles in DMEM + 10% FBS. For serum pre-incubation assay, nanoparticles were pre-incubated for 2 h at 37 °C in 5% glucose supplemented with 10% FBS. Then cells were incubated for 1.5 h with the nanoparticles in serum-free DMEM. After the addition of DMEM + 20% FBS (10% FBS final), cells were further incubated for 24 h and finally lysed for CyclinB1 western blotting detection.

For microscopy assay (spinning Disk), 400,000 cells were seeded 24 h before imaging into a Fluoro Dish from World Precision Instruments (tissue culture dish with cover glass bottom, dish diameter = 35 mm/glass diameter = 23 mm/glass thickness 0.17 mm). Before microscopy imaging, cells were washed and covered with 1600 µl of complete medium. 400 µl of nanoparticles (Atto633-RICK:siRNA-Cy3b; R = 20), formed as previously described in 5% glucose water, were directly added on the cells at the very beginning of imaging.

#### Measurement of cell cytotoxicity

Evaluation of cytotoxicity induced by the nanoparticles was performed using Cytotoxicity Detection Kit^Plus^ (LDH, Roche Diagnostics) on 50 µl of supernatant, by following the manufacturer instructions.

#### Luciferase reporter gene silencing assay

The evaluation of siRNA delivery using the different vectors was carried out by measuring the remaining luciferase firefly (FLuc) and luciferase *Renilla* (RLuc) activity in cell lysates. Briefly, after 48 h, the medium covering the cells was carefully removed and replaced by 50 µl of 0.5× Passive Lysis Buffer (PLB; Promega). After 30 min of shaking at 4 °C, plates containing the cells were centrifuged (10 min, 1800 rpm, 4 °C) and 5 µl of each cell lysate supernatant were finally transferred into a white 96-well plate. FLuc and RLuc activities were quantified using a plate-reading luminometer (POLARstar Omega, BMG Labtech), measuring light emission over a 2 s reaction period immediately after injections of 100 µl of half-diluted Dual Luciferase Assay Reagents (Promega) per well. The results were expressed as percentage of relative light units (RLU) in non-treated cells (%FLuc and %RLuc), then normalized on %RLuc to obtain the Relative Luc Activity (%FLuc/%RLuc).

#### Western blotting

Transfected cells washed in PBS, and lysed in buffer 150 mM sodium chloride, 1.0% Triton X-100, 0.1% SDS (sodium dodecyl sulphate), 50 mM Tris pH 8.0 including protease inhibitors (SigmaFAST). Cells were incubated for 5 min on ice with 130 µl/24-well lysis buffer. Thereafter, cells were scrapped and transferred in a 1.5 ml tube. After 5 min on ice, the cell lysates were centrifuged (10 min, 16,100*g*, 4 °C), supernatants were collected and protein concentrations were determined using the Pierce BCA Protein Assay (ThermoFisher). Cell extracts (0.25–0.375 μg/µl) were separated by 4–20% Mini-PROTEAN^®^ TGX™ Precast Gel (Bio-Rad). After electrophoresis, samples were transferred onto Trans-Blot^®^ Turbo™ Mini PVDF Transfer membrane (Bio-Rad). As antibodies (all from Cell Signaling), we used anti-cyclin B1 mouse mAb V152, anti-Vinculin rabbit mAb E1E9V, anti-mouse IgG HRP and anti-rabbit IgG HRP. Blots were revealed with the Pierce ECL plus Western blotting substrate (ThermoFisher) on an Amersham imager 600 (GE Healthcare Life Science). The signal intensities of the blots were quantified by Image J.

### Membrane interaction and internalization of CPP:siRNA nanoparticle

#### GUV imaging by confocal microscopy

For the microscopy experiments, the GUVs were placed into a glass bottom cell culture dish (Greiner bio-one) and incubated with 400 nM Atto633-RICK:20 nM siRNA-Cy3b. Confocal images of GUVs were immediately obtained with an inverted LSM780 multi-photon microscope (Zeiss). The obtained confocal images were projected and treated with the software ImageJ.

#### Liposome leakage assay

Large unilamellar vesicles (LUV) reflecting the plasma membrane were prepared as described in detail in Additional file 1. Leakage was measured as an increase in fluorescence intensity upon addition of the CPP or CPP nanoparticle (500 nM final CPP concentration) to 2 ml of LUVs (100 μM) in buffer (20 mM HEPES, 145 mM NaCl, pH 7.4). 100% fluorescence was achieved by solubilizing the membranes with 0.1% (v/v) Triton X-100 resulting in the completely unquenched probe.

#### Spinning disk confocal microscopy

To study the entrance of the NPs inside living cells, we used an inverted microscope (Nikon Ti Eclipse) coupled to a spinning disk (ANDOR) system. An excitation laser was used to illuminate a 60× (1.4 numerical aperture) oil immersion objective. The scanning system microscope was equipped with a motorized stage ASI allowing sample scanning in x, y and z direction. The spinning disk head was a Yokogawa CSU-X1 with 3 dichroic: mono, dual or quad-band filter. To visualize the fluorescence, we used as emission filter a 4 band pass filter (QUAD dichroic) center around 405, 488, 561 and 640 nm. Cells were maintained at 37 °C by a cage incubator (Okolab) all along the 2 h of measurements. Details concerning the acquisition parameters were provided in Additional file 1. No emission bleed through was observed between the different channels observed with these acquisition parameters. Emission photons were collected on an EM-CCD camera (iXon Ultra) and images were recorded every 2 for 120 min and projected with the software Andor IQ3. These acquisition parameters provide an image of 512 × 512 pixels with a pixel size of 0.15 µm. The image treatment was performed with the Imaris software for a 3D reconstruction.

## Results and discussion

### Structural characterization

**Fig. 1:**
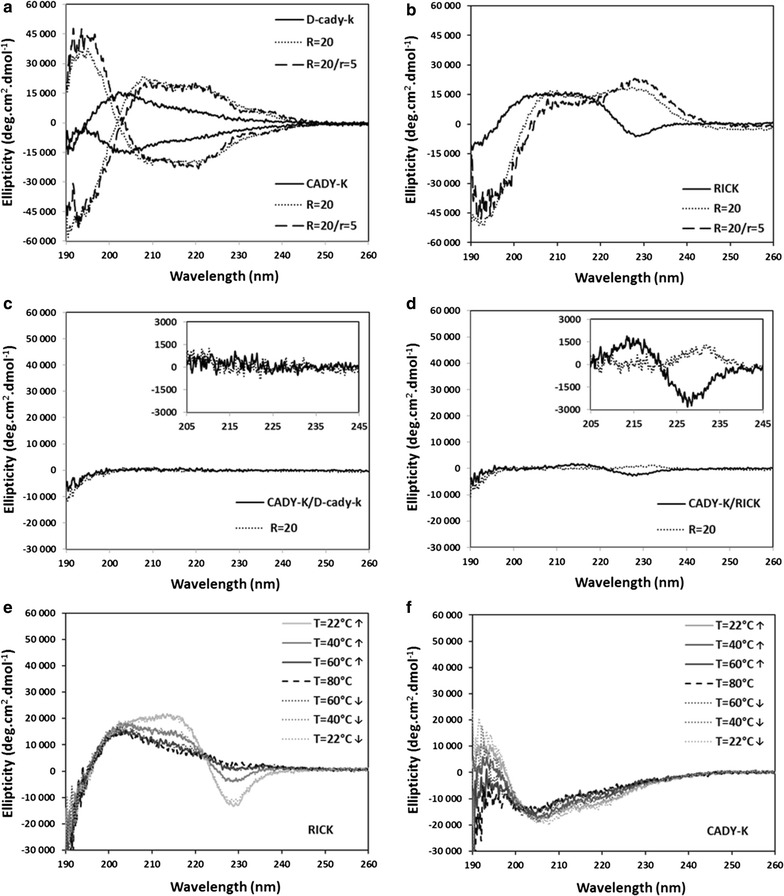
Structural characterization of RICK and isoforms. **a**, **b** Circular dichroism (CD) profiles of CADY-K, d-cady-k and RICK in solution, associated to siRNA anti-FLuc at a 20/1 molar ratio (R = 20) and in the presence of LUVs composed of DOPC/SM/Chol (2:2:1) and at a final lipid/peptide ratio of 5 (r = 5). **c**, **d** CD spectra of CADY-K/d-cady-k and CADY-K/RICK mixtures in solution at an equimolar ratio and in the presence of siRNA at a final peptide:siRNA ratio R = 20. **e**, **f** Effects of heating/cooling cycle from 22 to 80 °C on CD spectra of RICK and CADY-K. CD signals are expressed in Ellipticity

As already described for the CADY peptide and its analogues, the conformational state has a strong importance for its ability to interact on one hand with the siRNA cargo and on the other hand with phospholipids constituting the main component of biological membranes [25, 31, 32]. Thus, the structural state of RICK was investigated by circular dichroism (CD) and compared to those obtained for its enantiomers CADY-K and d-cady-k. Secondary structure was determined when peptide is free in solution, mixed with siRNA at a peptide:siRNA molar ratio of R = 20, or when liposomes are added to the peptide:siRNA mix at a lipid/peptide ratio of r = 5 (Fig. 1a, b).

As previously identified, CD profile of free CADY-K has a minimum at 203 nm and a weak shoulder at 220 nm (Fig. 1a), suggesting a mainly random coil conformation with few helical contribution [25]. Similarly, in the presence of siRNA CADY-K adopts a right-handed α-helical structure (maximum at 191 nm, minima at 210 and 220 nm) which is not impacted by addition of zwitterionic large unilamellar vesicles (LUVs) made of DOPC/SM/Chol (2:2:1). As expected, CD spectra of d-cady-k are clearly mirror images of CADY-K profiles (Fig. 1a). The spectra of free d-cady-k, associated with siRNA and in the presence of liposomes have bands centered at the same wavelength of CADY-K signals. The conversion of l- to d-amino acids only induces an inversion of bands intensity while maintaining similar absolute amplitude of the l-form, resulting in a perfect symmetry as already observed for some antimicrobial peptides [33–35]. This indicates that the enantiomers CADY-K and d-cady-k have exactly the same conformational behavior: while free peptides are mainly disordered in solution, CADY-K and d-cady-k adopt right-handed and left-handed helical structure, respectively, in the presence of siRNA.

Surprisingly CD spectra of RICK show two main differences (Fig. 1b). Firstly, the CD spectrum of free peptide consists in less defined extrema. A decrease of the signal around 190 nm does not reflect a net minimum and the positive band between 202 and 220 nm might be considered as a wide maximum suggesting a mix of several structures hardly identifiable/assignable. Secondly, contributions of close tryptophan/tryptophan interactions are clearly observed through the band at 228 nm which is negative for d-amino acids [36, 37]. These contributions suggest a cluster of tryptophans within the peptide sequence, as observed for tryptophan zipper peptides [38]. The addition of siRNA induces a net minimum at 192 nm with a significant maximum at 208 nm which suggest a main left-handed helical structure, although the critical region at 222 nm seems to be hidden by a pronounced positive band at 228 nm. The negative tryptophan band turns to a positive one, indicating a conformational change with a reorganization of the tryptophan residues.

In order to further understand structural differences observed for RICK, we studied the CD spectra of equimolar mixtures of enantiomers CADY-K/d-cady-k and CADY-K/RICK. Indeed, considering that all peptides might not interfere each other in their way to interact with siRNA and LUVs, investigations of these mixtures should underline structural variations between isoforms compared to the parent peptide CADY-K. Analyses of CADY-K/d-cady-k mixtures, with or without siRNA, result in the absence of any CD signal (Fig. 1c). The mean molar ellipticity does not vary and remains at zero level (Fig. 1c, insert; Additional file 1: Figures S1), as expected for a mix of the l- and the d-enantiomer (compensation of each contribution) [39]. These observations clearly confirm the similar structure of CADY-K and d-cady-k and furthermore underline the absence of specific interactions between both isomers. In contrast, analyses of CADY-K/RICK mixtures reveal a slight inflection centered at 228 nm, the tryptophan region, which varies in the presence of siRNA (Fig. 1d, inset; Additional file 1: Figure S1). Although weak, this effect suggests that the *retro*-*inverso* peptide contains different tryptophan/tryptophan interactions upon its complexation to siRNA.

This structural particularity of RICK was then analyzed through thermal unfolding to evaluate the influence of the temperature on these hydrophobic interactions. Applying heating/cooling cycle from 22 to 80 °C, we could detect several variations in the CD profiles of RICK (Fig. 1e), whereas no significant change was observed for CADY-K (Fig. 1f). An increase of temperature above 40 °C induced a denaturation of RICK mainly characterized by a loss of amplitude at 215 and 228 nm. These modifications form an isodichroic point at 220 nm suggesting an equilibrium between two structural states of RICK: folded at low temperature versus denatured at high temperature. In addition the CD profile of heated RICK (60/80 °C) is superimposable with the CD spectrum of d-cady-k at 25 °C, suggesting the same random coil conformation (Additional file 1: Figure S2). In conclusion, these analyses reveal that RICK adopts a structure which is sensitive to thermal unfolding. Similar thermal unfolding, associated to a loss of 228 nm contribution, was described for tryptophan zipper β-hairpins (Trpzip) based on the unfolding of the zipper [40, 41]. Thus, in a similar manner, our data suggest that tryptophan residues of RICK establish tryptophan/tryptophan interactions forming a kind of tryptophan zipper. Finally, these interactions change in the presence of siRNA, leading to a conformational change in a left-handed helix with peptide/siRNA interactions.

### Nanoparticle characterization

**Fig. 2:**
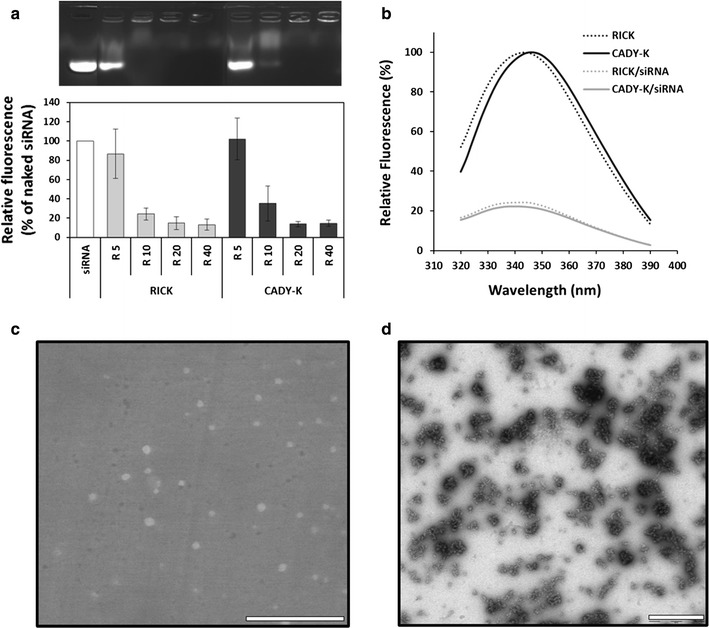
Characterization of RICK:siRNA nanoparticles. **a** Pre-formed RICK:siRNA and CADY-K:siRNA complexes were analyzed by electrophoresis on agarose gel (1% wt/vol) stained with GelRed. Data represent: mean ± SD, with n = 3. **b** Intrinsic tryptophan fluorescence emission spectra of RICK and CADY-K in 5% glucose and in the presence of siRNA at a peptide:siRNA molar ratio of R = 20. **c**, **d** Environmental scanning electron microscopy (ESEM) and transmission electron microscopy (TEM) images of RICK:siRNA nanoparticles in ultra-pure water at a peptide/siRNA molar ratio of R = 20. *Scales bars* correspond to 5 µm and 500 nm for ESEM and TEM, respectively

Peptide-based nanoparticles (PBN) involve formation of stable particles through interactions between carrier peptide and the cargo molecule [17, 19, 21, 23]. The conformational changes detected by CD suggested that RICK interacts with siRNA. To assess complexation of siRNA, we investigated the formation of RICK:siRNA complexes by agarose shift assay (Fig. 2a). siRNA alone migrated into the agarose gel but when complexed with CPPs, the peptides prevented oligonucleotide migration in a molar ratio-dependent manner. For better quantification, the fluorescence signals of the CPP:siRNA complexes are represented relative to the signal intensity of siRNA alone (=100%). Although a slight difference could be noticed with CADY-K at a molar ratio R = 10, both peptides (RICK and CADY-K) were clearly able to complex siRNA in a similar manner with optimal complexation at peptide:siRNA molar ratios of R = 20 and R = 40 (Fig. 2a).

We then monitored the intrinsic tryptophan fluorescence of RICK and CADY-K with or without siRNA. Fluorescence emission spectra of free peptides revealed that CADY-K was characterized by a maximum of fluorescence intensity centered at 348 nm, corresponding to tryptophan usually exposed to the aqueous solution [42] (Fig. 2b). In contrast RICK had a maximum of fluorescence at 345 nm, which was slightly shifted to lower wavelength (blue shift) compared to CADY-K. This suggested a more hydrophobic environment for the tryptophan of RICK, such as the tryptophan/tryptophan cluster observed by CD. In the presence of siRNA, at a final peptide:siRNA molar ratio of R = 20, a similar change was detected in the spectra of both peptides. A net decrease of fluorescence intensity and a shift of the maximum of fluorescence to 340 nm were observed (Fig. 2b). Previous investigations indicated that the intrinsic tryptophan fluorescence of peptides might undergo a strong quenching in the presence of nucleic acids based on aromatic stacking effects [16, 43]. This aromatic stacking process might be also associated to the blue shift, from 348 to 340 nm for CADY-K and from 345 to 340 nm for RICK, which suggests a more hydrophobic environment of the tryptophan. Our results were in agreement with the fact that short peptides are able to undergo tryptophan fluorescence quenching when interacting with single-stranded nucleic acids as well as DNA duplex [44–46]. For example, the strong binding of the KWGK peptide to a 21-mer duplex involves intercalation and stacking interactions of the tryptophan with the oligonucleotide GC regions. In this context, the quenching of tryptophan fluorescence was due to an electron transfer from indole of the tryptophan side chain (in the excited state) to purine and pyrimidine bases [46]. Taken together, these results suggested that the siRNA was able to destabilize the RICK tryptophan-zipper-like structure (loss of Trp/Trp interactions) in favor of the RNA bases/tryptophan interactions.

Colloidal features of RICK:siRNA complexes were characterized and compared to CADY-K and d-cady-k. Size and homogeneity were determined for each complex (5% glucose, R = 20). Dynamic light scattering (DLS) investigations enable to express particle size in intensity, volume or number [47]. Intensity measurements (%) revealed that RICK, CADY-K and d-cady-k formed peptide-based nanoparticles with diameter of ~100 nm with polydispersity index (PDI) of ~0.28 (Table 1; Additional file 1: Figure S3). Because it is well known that the diffused light intensity (%) is dependent of the measured particle size, we decided to evaluate also the size distribution based on number (%). Indeed, the percentage of intensity resulting from light diffused by bigger particles is one million fold stronger than those from small ones. Thus, signals of small particles are under evaluated because they are not visualized in size distribution based on intensity recording. Size distribution based on number (%) obtained using algorithms could give us a more accurate information about size distribution. This nanoparticle size representation suggests the presence of smaller nanoparticles in addition to those found by the intensity-based size distribution. These smaller particles have diameters of ~30 nm for RICK:siRNA and its isoforms (Table 1; Additional file 1: Figure S3). More importantly, both measured size values did not change significantly even after 72 h storage at 4 °C, which is in agreement with previous experiments with CADY or CADY-K [25, 48].

Charge surface of nanoparticles was also evaluated by zeta potential (ZP) measurements revealing similar values of ~40 mV for all three CPP:siRNA complexes in 5% glucose (Table 2). Same values were obtained in an aqueous solution containing 5 mM NaCl or in 5% glucose for the parental peptide CADY:siRNA [25, 48]. However, it could be interesting to evaluate ZP in culture media used during transfection (OptiMEM and serum free DMEM). Table 2 showed the ZP changes to more neutral values; however, the overall charge remained in the positive range, which is required for the cellular translocation. Only the addition of 50% serum induced a negative ZP of −12 mV [25] but this condition is not relevant to in vitro transfection conditions. Yet, we should note here that Lindberg et al. measured a negative ZP for CADY:siRNA. This difference with our findings could be due to many parameters such as formulation, siRNA length, molar ratio, concentration etc. [49].

**Table 2:** Zeta potential (ZP) of RICK, CADY-K and d-cady-k

ID	ZP (mV)		
	5% glucose^a^	OptiMEM	DMEM
RICK	40 ± 2	14 ± 3	14 ± 1
CADY-K	38 ± 1	13 ± 1	12 ± 3
d-Cady-k	40 ± 1	n.d.	n.d.

All CPP:siRNA complexes were formed at R = 20 using a siRNA concentration of 500 nM in an aqueous solution of 5% glucose, OptiMEM or DMEM

^a^ For zeta potential (ZP) assessment the solution was supplemented with 5 mM NaCl (OptiMEM and DMEM contained NaCl)

To obtain more details regarding their size but also their shape, RICK:siRNA nanoparticles were analyzed by environmental scanning electron microscopy (ESEM) and transmission electron microscopy (TEM). Several samples were prepared in ultra-pure water as the use of 5% glucose might interfere with the imaging. Analyses of ESEM samples revealed globular nanoparticles as already observed for CADY peptide [48]. Measurements of diameters indicated a mean size centered at 300 nm and ranging from 150 to 550 nm (Fig. 2c). These sizes were consistent with the different hydrodynamic diameters identified by intensity (%) measurements, suggesting a similar Gaussian size distribution.

To gain more details on the nanoparticle shape, we performed TEM measurements which allow a better resolution to measure small nanoparticles. TEM investigations revealed nanoparticles with a mean size of ~120 nm and also smaller nanoparticles with a mean diameter of ~21 nm (Fig. 2d; Additional file 1: Figure S4). Even if the TEM sample preparation could induce self-assembly phenomena, the obtained images are in good agreements with DLS results in size distribution based on number (%) and intensity (%) which highlighted the presence of small (~30 nm) and bigger (~100 nm) nanoparticles in solution (Table 1; Additional file 1: Figure S3).

These observations were in agreement with the nanoparticle formation of CADY:siRNA determined by molecular modeling or by atom force microscopy (AFM), as previously demonstrated [48]. We could hypothesize that RICK:siRNA nanoparticles were formed in the same manner: first the formation of ~20 nm spheres composed of RICK:siRNA (R = 20) which self-associate in a “beads necklace”-like manner to nanoparticles of ~100 nm. Such assembly could be also compared to the “large branching agglomerates” described for the parental peptide CADY in the presence of miRNA [50].

### Proteolysis resistance

In order to prevent proteolysis, peptides composed of l-amino acids are usually synthesized using their corresponding unnatural d-isomer. Because this replacement can induce a loss of biological activity due to a different side chain orientation, *retro*-*inverso* peptides—the d-amino acids in the reverse sequence of the naturally occurring l-peptides—can be used as a peptidomimetic approach.

**Fig. 3:**
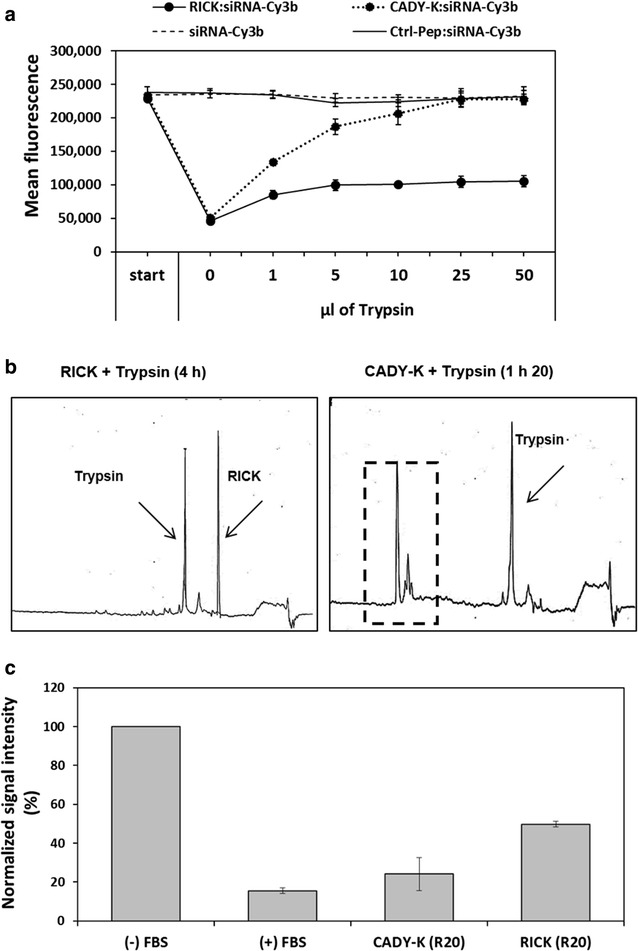
Analysis of the proteolysis resistance of the RICK peptide and the consequence on the PBN stability. **a** Fluorescence emission of siRNA-Cy3b alone or in the presence of RICK, CADY-K and a control peptide (Ctrl-pep) at a peptide molar ratio of R = 20 in the presence of increasing trypsin amounts. **b** HPLC chromatograms of RICK and CADY-K in the presence of Trypsin. The *dotted square* represent the degraded CADY-K. Degradation products are given in Additional file 1: Table S1. **c** siRNA recovery after serum incubation. Cy3-labeled siRNA alone or complexed to CADY-K or RICK (R = 20) were incubated with serum. siRNAs were extracted from the sample and visualized in an acrylamide gel. Resulting fluorescence intensities were normalized to untreated siRNA (−FBS)

In order to investigate the stability of RICK compared to CADY-K, we analyzed the siRNA release of formulated nanoparticles (CPP:siRNA-Cy3b, R = 20) by fluorescence spectroscopy after 24 h of incubation with increasing trypsin concentrations (Fig. 3a). At the beginning the mean fluorescence of the siRNA-Cy3b alone (start) is around 240,000. As expected, the fluorescence was quenched in the same way when RICK or CADY-K was added (mean fluorescence of ~50,000 equal to a 4.6-fold decrease). Control experiments with siRNA-Cy3b alone or in the presence of a peptide not able to form nanoparticles (Ctrl-Pep:siRNA) revealed no change in the fluorescence intensity during the whole assay.

In contrast, by adding an increasing amount of trypsin, we observed a progressive release of the siRNA of the previously formulated CADY-K:siRNA nanoparticles. At the highest trypsin concentration the initial siRNA fluorescence was completely restored, probably due to the total CADY-K degradation. Applying the same conditions to the RICK:siRNA nanoparticles, nearly no change in the mean fluorescence intensity was observed, confirming the stability of the complex in the presence of trypsin.

HPLC analyses were performed to evaluate the impact of trypsin on RICK and CADY-K peptide sequence. At a time point zero (t = 0), we observed two peaks on the HPLC spectra corresponding to the peptide and the trypsin. As expected, the HPLC chromatograms show that CADY-K was completely degraded within 80 min of trypsin incubation, whereas RICK is stable over time (4 h and more) (Fig. 3b). To confirm that the degradation products of CADY-K were due to trypsinization, we collected the corresponding HPLC fractions for MS/MS analysis (Additional file 1: Table S1).

Finally, we evaluated the efficient siRNA-Cy3 protection by RICK or CADY-K nanoparticle formation in the presence of serum. After a 24 h incubation at 37 °C, the siRNA was extracted from the sample preparation and quantified by Cy3 detection after acrylamide gel migration. The measured signal intensities were normalized to the conditions without serum (−FBS) (Fig. 3c). When the siRNA was directly incubated with serum (+FBS), 84% of the siRNA signal is lost, revealing its rapid degradation. In contrast, in the presence of CADY-K 24% of the siRNA was rescued, and more importantly, 50% when the siRNA was protected by RICK. Due to the *retro*-*inverso* properties, we obtained a twofold higher resistance to proteases for RICK compared to CADY-K.

In sum, we demonstrated through three approaches that RICK is more stable against proteolysis phenomena (trypsin or serum) compared to CADY-K. To our knowledge, only Seelig and Coll are working on *retro*-*inverso* CPP in a non-covalent strategy (riDOM:plasmid) [51, 52]. However, they did not look at trypsin or serum resistance of their peptide. Furthermore, only few publications comparing different enantiomers in terms of protease stability are available. For example, the SAP CPP enantiomers exhibit the same behavior in terms of cellular delivery but d-isomer showed better stability to proteases than the l-form [53], confirming our results.

### Membrane interaction and internalization of RICK:siRNA nanoparticles

**Fig. 4:**
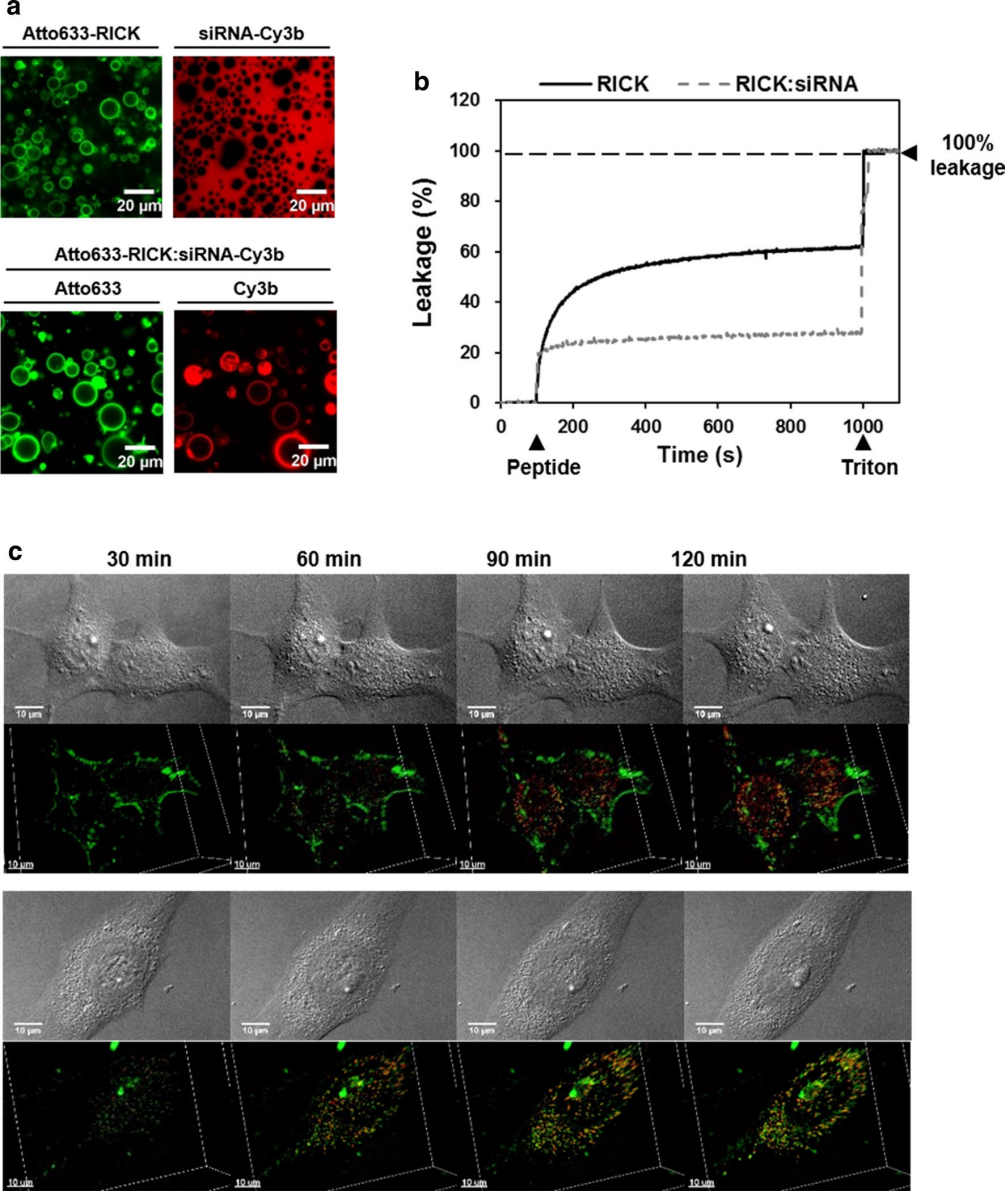
Membrane interaction and internalization of RICK:siRNA nanoparticles on GUVs, LUVs and living cells. **a** Representative fluorescence microscopy images of GUVs incubated with 400 nM Atto633-RICK or 20 nM siRNA-Cy3b alone or with the formulated nanoparticles (400 nM Atto633-RICK:20 nM siRNA-Cy3b). *Bars* represent 20 µm. **b** Comparison of the leakage properties of 500 nM RICK alone and 500 nM RICK:25 nM siRNA nanoparticles on LUVs [DOPC/SM/Chol (2:2:1)]. Peptides/nanoparticles were injected at 100 s and the Triton (positive control) at 1000 s (n ≥ 2). **c** Representative 3D confocal microscopy images of the Atto633-RICK:siRNA-Cy3b internalization in living U87 cell lines. Images were taken every 30 min during 2 h, to follow fluorescence entrance and repartition inside the cell. For the 3D representation, the images were treated with Imaris software. *Bars* represent 10 µm

Once we demonstrated the proteolysis resistance of RICK peptide, we looked at RICK behavior in the presence of lipid bilayers. Giant unilamellar vesicles (GUVs) are often used to mimic the cell plasma membrane in a simplified environment [54]. With size ranging from 1 to 100 μm, they provide good models to investigate interactions between lipid bilayers and non-lipid molecules such as CPPs or nanoparticles. The ability of nanoparticles to target, label, and/or penetrate lipid bilayers has resulted in great interest in correlating these phenomena with nanoparticle penetration into cells or even with nanoparticle-related cytotoxicity. To evaluate the interaction of RICK nanoparticles with lipid membranes, we decided to generate GUVs of simple composition using POPC in order to minimize phase separation phenomena during GUV formation. First, we incubated the GUVs with siRNA-Cy3b or with Atto633-RICK alone. As shown in Fig. 4a, the siRNA was homogeneously distributed in the surrounding solution without association to the lipid bilayer, whereas the labeled peptide immediately stuck to the GUV’s membrane. RICK peptide (positively charged) was naturally attracted by lipids (negatively charged), whereas, the siRNA (negatively charged) was rather repulsed.

When the GUVs were incubated with the nanoparticles Atto633-RICK:siRNA-Cy3b, we noticed that both molecules stuck predominantly to the membrane (Fig. 4a). Furthermore, when the GUVs were first incubated with the siRNA-Cy3b and thereafter the non-labelled RICK peptide was added to this mixture (to avoid Cy3b quenching), addition of the peptide led to a fluorescent profile change. Indeed, we could observe a reorganization of siRNA-Cy3b that progressively associates in a dotted pattern to the GUV’s membranes (Additional file 1: Figure S5). The irregular dotted pattern was probably due to an unregular distribution of unlabeled RICK peptide and RICK:siRNA-Cy3b nanoparticles or to a different nanoparticles formulation in this assay. The obtained results demonstrated that the RICK peptide was able to recruit siRNA molecules to the GUV’s membrane by its dual interaction with siRNA and lipid membranes. Attraction of RICK for lipid membranes was further confirmed by Langmuir adsorption at an air–water interface (CMC ~100 nM, Πsat ~35 mN/m) (Additional file 1: Figure S6A) as well as by the enhancement of intrinsic tryptophan fluorescence emission associated to a 10 nm blue-shift in the presence of an excess of liposomes (Additional file 1: Figure S6B).

In order to evaluate the lipid membrane interaction and/or transduction of the nanoparticles, we performed leakage assays using LUVs with a complex lipid composition [DOPC/SM/Chol (2:2:1)] reflecting the plasma membrane. In the absence of peptides (or nanoparticles), no leakage was observed based on the low permeability of the phospholipid vesicle membrane to the fluorophore/quencher mix (Fig. 4b). Addition of peptide alone on the LUVs induced a significant increase in fluorescence revealing an important leakage. At the endpoint (after 15 min incubation), we obtained a leakage of 60% compared to the Triton (positive control). In contrast, the RICK:siRNA nanoparticle leakage was twofold weaker (28%) than the one observed for the peptide alone (60%). This is in agreement with the fact that in the RICK:siRNA complexes one part of the peptide interacts with the siRNA and the other part with the lipids.

Finally, we looked at the cellular internalization of the nanoparticles in living U87 human glioblastoma cells using spinning disk confocal microscopy (Fig. 4c; movie in Additional file 2). One of the advantage of using this technique was the possibility of a 3D reconstruction of the cell and a better understanding of nanoparticles distribution inside the cell. Furthermore, fluorescence quantification of siRNA-Cy3b and RICK-Atto633 allowed us to determine nanoparticles behavior once they reached the cell and crossed the plasma membrane. Based on this quantification, in the first 30 min, we could observe the progressive internalization of both RICK and the siRNA. After 90 min, the fluorescence patterns of both molecules were uniform in the cytoplasm reaching a maximum at 120 min. The fluorescence profile was exclusively cytoplasmic, no nanoparticles were found inside the nucleus, but it seems that the siRNA accumulated around the nucleus (at ~90 min). In some case, we could also visualize the accumulation of Atto633-RICK at the plasma membrane over the whole incubation period (2 h), which indicated the strong affinity of RICK peptide for lipids, while siRNA was never found at the plasma membrane. Furthermore, longer incubation (>24 h) of the nanoparticles did not give rise to higher cytosolic accumulation of Atto633-RICK or siRNA-Cy3b (data not shown).

In conclusion, we could state that nanoparticles had no lytic properties (no GUV or LUV destruction) and that the amphipathic peptide did not induce any membrane perturbation (no inner vesicle or tube formation) as reported by Ayala-Sanmartin and coll. for R9 and RW9 [55]. Finally, the RICK:siRNA internalization occurred immediately after applying the nanoparticles on cells. A minimal incubation time of 90 min should be sufficient to deliver the siRNA and then to induce a biological activity. Internalization mechanism of our RICK-based PBN is estimated to be through direct translocation based on the positive ZP value in transfection medium (Table 2) as well as on previous microscopic experiments with the parental CADY peptide showing no co-localization with endosome markers [56].

### Cellular evaluation OF RICK:siRNA nanoparticles

A luciferase gene silencing assay, in combination with an LDH cytotoxicity assay, was used to compare the efficiency of siRNA-loaded nanoparticles formulated with RICK, CADY-K and d-cady-k. For this purpose, we used an U87 cell line stably transfected with two plasmids containing firefly (FLuc) and *Renilla* (RLuc) luciferase reporter genes under transcriptional control of their own constitutive Cytomegalovirus (CMV) promoter. This cell line allowed us to knock-down FLuc (siFLuc) and to use RLuc as an internal control for the normalization (see scheme in Additional file 1: Figure S7). Based on our previous work [25] and on current structural characterization, peptide:siRNA nanoparticles have been formulated in 5% glucose, R = 20, with a siRNA targeting FLuc (siFLuc) at three different concentrations (5, 10 and 20 nM). In addition, a scrambled siRNA version (siSCR) was used as negative control. All the FLuc activities were normalized to the RLuc levels and to non-treated cells and then plotted as Relative Luc Activity (%).

**Fig. 5:**
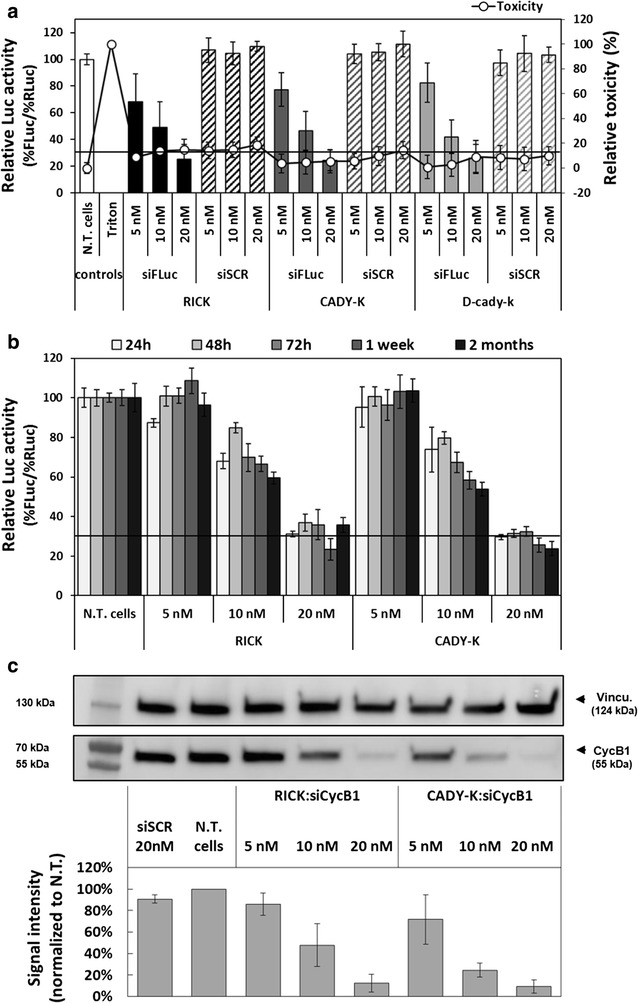
Cellular evaluation of RICK:siRNA nanoparticles. **a** Relative Luc activity (%FLuc/%RLuc) and relative toxicity (LDH quantification) after transfection with RICK-, CADY-K- and d-cady-k-based complexes in U87-FLuc-RLuc cells. siFLuc and siSCR correspond to siRNA anti-firefly Luciferase and its scrambled version, respectively. **b** Evaluation of RICK:siLuc and CADY-K:siLuc inhibiting efficiency after different PBN storage duration. Relative Luc activity (%FLuc/ %RLuc) was measured after PBN storage of 24, 48, 72 h, 1 week and 2 months. **c** Gene silencing on the endogenic protein cyclin B1. *siSCR* scrambled siRNA, *N.T.* non-treated cells, *siCycB1* siRNA anti-Cyclin B1, *Vincu.* Vinculine

As shown in Fig. 5a, the relative LUC activity (%) recorded for RICK:siRNA, CADY-K:siRNA and d-cady-k:siRNA nanoparticles revealed a specific and similar dose-dependent knock-down of FLuc reaching ~75% of inhibition at the highest siRNA concentration (20 nM). These knock-downs were specific for the used nanoparticles, because the same nanoparticles formulated with the siSCR show a Luc activity similar to the non-treated cells. For CADY-K, these results were in agreement with those reported previously on Neuro-2a-Luc^+^ and B16-F10-Luc^+^ cells [25].

With regard to cytotoxicity, all values were close to those measured for untreated cells (0%) and not higher than 20% (Fig. 5a). In this context, cytotoxicity evaluation was an important feature to avoid false-positive results because toxic effect would have an impact on both luciferase activities. Under the applied conditions (5000 cells per well), we observed at siRNA concentrations higher than 60 nM (=1.2 µM RICK concentration) that the normalized results were no longer coherent based on the unspecific knock-down of RLuc (data not shown).

In parallel, stability of the PBNs in the course of storage was investigated over the time with regard to future clinical applications. Although the DLS investigations have already indicated that nanoparticles were stable and conserved the same colloidal features after 72 h at 4 °C, we decided to control the FLuc knock-down efficiency of siRNA-loaded RICK PBN over the time. A stock solution of RICK:siRNA and CADY-K:siRNA complexes (5% glucose, R = 20) were formulated, stored at 4 °C and aliquots were used for luciferase screening after 24, 48, 72 h, 1 week and 2 months (Fig. 5b). Results revealed no significant difference between the dose-dependent knock-down of FLuc, whatever the moment of the luciferase assay. As internal control, “freshly” prepared RICK:siRNA and CADY-K:siRNA nanoparticles were used to validate the obtained results. In all cases (fresh versus stored solution), the knock-down efficiency seemed to be slightly lower (~70% for 20 nM, ~35% for 10 nM and ~5% for 5 nM siRNA) than previously observed (~75% for 20 nM, ~55% for 10 nM and ~22% for 5 nM siRNA) (Fig. 5b and data not shown). Other samples of RICK:siRNA and CADY-K:siRNA nanoparticles evaluated after a long storage period (6 months and 1 year at 4 °C) confirmed that these nanoparticles were stable over the time and induced the same knock-down activity (data not shown).

To confirm the efficiency of the RICK:siRNA nanoparticles, gene silencing was also undertaken on the endogenic protein cyclin B1 which is known to be deregulated in most common cancers [57–60] instead of an overexpressed system (luciferase). After an optimization step (cell number per well and siRNA concentration), the knock-down efficiency of RICK:siRNA and CADY-K:siRNA nanoparticles (5% glucose, R = 20) were evaluated in a dose-dependent manner (5, 10 and 20 nM siRNA). The resulting protein expression (blot signal intensities) were first normalized to the loading control vinculin and thereafter to the non-treated condition (N.T cells) (Fig. 5c). As expected, we clearly observed a specific knock-down of Cyclin B1 (no effect using siSCR) for both types of nanoparticles. The only difference consisted in the different systems we used: in the overexpressed system, 20 nM siRNA were required for an 80% reduction of FLuc expression in 5000 cells whereas only 10 nM were necessary for the same percentage of Cyclin B1 knock-down in 75,000 cells. That discrepancy was directly due to the CMV-dependent overexpression of FLuc which is probably more than sevenfold higher than the endogenic expression of the Cyclin B1.

In a similar glioma cell line, Youn and coll. found ~40% reduction in luciferase expression using a myristic acid conjugated-CPP (transportan; TP) equipped with a transferrin receptor-targeting peptide (myr-TP-Tf) complexed with siRNA-FLuc, suggesting a high activity of RICK-based nanoparticles [61].

**Fig. 6:**
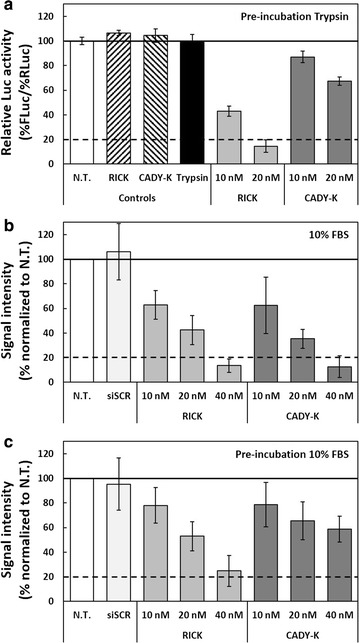
Evaluation of nanoparticles in the presence of trypsin or serum. **a** Relative Luc activity (%FLuc/%RLuc) after transfection with RICK- and CADY-K-based nanoparticles in U87-FLuc-RLuc cells after pre-incubation with trypsin. siFLuc and siSCR correspond to siRNA anti-firefly Luciferase and its scrambled version, respectively. **b** Evaluation of RICK:siCycB1 and CADY-K:siCycB1 inhibiting efficiency after transfection in the presence of medium with 10% serum. **c** Evaluation of RICK:siCycB1 and CADY-K:siCycB1 inhibiting efficiency after pre-incubation of nanoparticles 2 h in 10% serum at 37 °C before transfection. *siSCR* scrambled siRNA, *N.T.* non-treated cells, *siCycB1* siRNA anti-Cyclin B1

Finally, gene silencing abilities of RICK- and CADY-K-based nanoparticles were investigated after treatment with trypsin and through incubation with serum in order to evaluate biological activity for both peptides in proteolytic conditions. An overnight pre-incubation of nanoparticles in trypsin induced a net reduction of the FLuc knock-down efficiency for CADY-K compared to RICK (Fig. 6a). Only a ~30% decrease of FLuc activity was observed for CADY-K at 20 nM siRNA, compared to the 75% inhibition observed without trypsin treatment. In contrast, the level of inhibition observed for RICK remained similar to those obtained without trypsin treatment, i.e. ~60 and ~80% of FLuc silencing for 10 nM and 20 nM siRNA, respectively (Figs. 5a, 6a). These results clearly emphasized the resistance of RICK to proteases as shown previously by HPLC/MS (Fig. 3b; Additional file 1: Table S1).

In a similar manner, we addressed the effect of serum on nanoparticles through the monitoring of CyclinB1 knock-down. The presence of 10% FBS during the transfection induced a slight decrease of silencing efficiency, characterized by the necessity of using a twofold higher siRNA concentration (10, 20 and 40 nM instead of 5, 10 and 20 nM) to reach the same percentage of CyclinB1 inhibition in serum-free transfection media (Fig. 6b, compared to Fig. 5c). This is probably due to the interaction of the serum proteins with the nanoparticles (as shown by ZP changes) resulting in a reduced internalization which is translate by a lower knock-down activity. However, the CyclinB1 knock-down was the same for RICK-and CADY-K-based nanoparticles (inhibition of ~40, ~60 and ~90% of the protein expression for 10, 20 and 40 nM, respectively), suggesting the absence of any difference between both peptides in these conditions. We hypothesized that this phenomenon could be due to the fact that both nanoparticles were rapidly internalized (within minutes), thus strongly limiting degradation by proteases.

To simulate an in vivo administration (blood circulation before internalization), we performed a pre-incubation with 10% FBS (2 h at 37 °C before transfection). Under this condition, compared to RICK, a significant decrease of inhibition efficiency was observed for CADY-K (Fig. 6c). Comparing the nanoparticles loaded with 40 nM siRNA, the induced CyclinB1 knock-down by RICK:siRNA was 2.4-fold higher than the one obtained with CADY-K:siRNA (~25 and 60% for RICK:siCycB1 and CADY-K:siCycB1, respectively). This latter result confirmed the more pronounced stability for RICK in serum, as already observed for other d-isoform peptides [53].

All this data confirmed the high potential of RICK-based nanoparticles as a promising tool for siRNA delivery in vitro and in vivo.

## Conclusions

In this report, we present the development and the characterization of a new peptide-based nanoparticle for siRNA cellular delivery. Based on previously reported secondary amphipathic peptide CADY-K [25], we designed the RICK peptide by combining the use of d-amino acids to the inversion of the peptide sequence. Our results revealed that this *retro*-*inverso* peptide RICK retained the main unique properties of CADY-K such as conformational versatility, formation of RICK:siRNA nanoparticles and remarkable efficacy in siRNA cellular delivery. Interestingly, in contrast to other *retro*-*inverso* peptides [51, 62], we observed a structural difference concerning the tryptophan cluster between CADY-K (l-isomer), d-cady-k (d-(isomer) and RICK, highlighting the important role of this amino acid. Moreover, because tryptophan has emerged as an important amino acid for membrane translocation [63, 64], a tryptophan clustering is a potential feature that should be taken into account for future PBN designs.

The main advantage of RICK-based nanoparticles lays in resistance to enzymatic degradation and the resulting protection of the siRNA. RICK-based nanoparticles are able to rapidly internalize siRNA into cells and to induce the inhibition of gene expression at a low dose (~75% knock-down of overexpressed luciferase and ~80% of endogenous CyclinB1 with RICK:20 nM siRNA) without any significant cytotoxicity. In the presence of serum, the activity is maintained by doubling the siRNA concentration (80% of CyclinB1 knock-down for RICK:40 nM siRNA). Compared to other CPP-based siRNA carriers, the measured knock-down effect is remarkable because in many cases higher siRNA concentrations are required for a less important knock-down efficiency [20, 65–67]. More importantly, RICK nanoparticles preserve the activity in proteolytic conditions (trypsin or serum pre-incubation) compared to CADY-K.

In summary, our results underline that RICK-based nanoparticles have the same biophysical properties (structure, size, ZP etc.) and the same biological in vitro efficiency (siRNA-induced knock-down) in serum-free or serum containing medium. However, RICK-based nanoparticles have the outstanding property to avoid siRNA degradation based on its own proteolytic stability as demonstrated by pre-incubation with trypsin and serum. This is an important prerequisite for its in vivo application since longer blood circulation is required. In parallel, we already consider the PEGylation of RICK to further improve RICK-based nanoparticle application in vivo [68].

## Additional files

**Additional file 1.** Supplementary information about circular dichroism analyses of RICK and isomers mixtures, characterization of peptide-based nanoparticles by DLS and TEM, mass spectra analyses of CADY-K, characterization of membrane interactions by fluorescence microscopy and principle of the Dual luciferase evaluation.

**Additional file 2.** Cellular internalization of siRNA-Cy3b (red dots)/RICK-Atto633 (green dots) nanoparticles over 2 hours by confocal microscopy.

## Abbreviations

CPP: cell-penetrating peptide
PBN: peptide-based nanoparticle
siRNA: short interfering RNA
LUV: large unilamellar vesicles
GUV: giant unilamellar vesicles
FBS: fetal bovine serum
CD: circular dichroism
DLS: dynamic light scattering
PDI: polydispersity index
ZP: zeta potential
ESEM: environmental scanning electron microscopy
TEM: transmission electron microscopy
