# Supplementary material for: A retro-inverso cell-penetrating peptide for siRNA delivery
Source: J Nanobiotechnology. 2017 Apr 28;15:34. doi: 10.1186/s12951-017-0269-2 (PMC5410048; doi:10.1186/s12951-017-0269-2)
Supplement: Supplementary file 1 — Additional file 1. Supplementary information about circular dichroism analyses of RICK and isomers mixtures, characterization of peptide-based nanoparticles by DLS and TEM, mass spectra analyses of CADY-K, characterization of membrane interactions by fluorescence microscopy and principle of the Dual luciferase evaluation. [file 12951_2017_269_MOESM1_ESM.pdf]

Additional material

Title: A *retro-inverso* cell-penetrating peptide for siRNA delivery

Anaïs Vaissière[1*], Gudrun Aldrian[2*], Karidia Konate[1*], Mattias F. Lindberg[1], Carole Jourdan[1], Anthony Telmar[1], Quentin Seisel[1], Frédéric Fernandez[3], Véronique Viguier[3], Coralie Genevois[4], Franck Couillaud[4], Prisca Boisguerin[1], Sébastien Deshayes[1#]

Affiliations:

1 - Centre de Recherche de Biologie cellulaire de Montpellier, UMR 5237 CNRS, 1919 Route de Mende, 34293 Montpellier, France

2 - Sys2Diag, UMR 9005-CNRS/ALCEDIAG, 1682 Rue de la Valsiere, 34184 Montpellier, France

3 - Université de Montpellier, Place Eugène Bataillon, 34095 Montpellier, France

4 - EA 7435 IMOTION (Imagerie moléculaire et thérapies innovantes en oncologie), Université de Bordeaux, 146 rue Leo Saignat, 33076 Bordeaux, France

# Corresponding author: sebastien.deshayes@crbm.cnrs.fr

This PDF file includes:

- Supplementary material concerning the Materials and Methods section
- Table S1
- Figures S1 to S7.

Establishment of the U87-CMV/FLuc-CMV/RLuc cell line

DNA construct: The CMV/Rluc fragment was removed by enzymatic restriction from the pRL-CMV vector (Promega, Madison, WI, USA) and inserted into the pcDNA6.2 vector (Invitrogen, Carlsbad, CA, USA) to generate pcDNA6.2-CMV/Rluc vector.

Cell line generation: The U87-CMV/Fluc cell line was previously described (Fortin et al., 2012) and was further transformed by pcDNA6.2-CMV/Rluc vector using Transfast™ transfection reagent (Promega) according to the manufacturer's instructions. Blasticidin (2 µg/ml; PAA, Piscataway, NJ, USA) allowed for selection of the U87-CMV/Fluc-CMV/Rluc cell line.

Liposome leakage assay

Large unilamellar vesicles (LUV) were prepared by removing organic solvent (evaporation for 45-60 min at 80°C). Thereafter, the lipids were hydrated in buffer (20 mM HEPES, 75 mM NaCl, pH 7.4) containing 12.5 mM ANTS fluorescent dye (8-aminonaphthalene-1,3,6-trisulfonic acid, disodium salt; Invitrogen) together with 45 mM DPX quencher (p-xylene-bispyridinium bromide; Invitrogen). The suspension was vigorously agitated with a Vortex (30 s), freeze-thawed 5 times and then extruded 21 times through two stacked 100 nm polycarbonate filters (Nucleopore, Whatman). Free dye and quencher were removed by gel filtration on a PD-10 desalting column (Amersham Biosciences). The following lipids (Avanti Polar Lipids) were used to mimic plasma membrane composition: dioleoylphosphatidylcholine (DOPC) / sphingomyeline (SM) / cholesterol (CHOL) (4:4:2; mol/mol/mol). LUV concentration was accessed using the LabAssay Phospholipid kit (Wako) as described by the manufacturer and LUV mean size was determined by Dynamic light scattering (NanoZS, Malvern).

Fluorescence leakage assay was measured on a PTI spectrofluorometer at 25°C (Ex = 360 nm $\pm$ 3 nm; Em 530 nm $\pm$ 5 nm). In details, LUVs were diluted in 1 ml buffer (20 mM HEPES, 145 mM NaCl, pH 7.4) to a final concentration of 100 µM. To access the background fluorescence, the LUVs alone were measured

during 100 seconds. Thereafter, leakage was measured as an increase in fluorescence intensity upon addition of RICK or RICK-based nanoparticle (500 nM final RICK concentration) during the following next 900 seconds (15 min). Finally, 100% fluorescence was achieved by solubilizing the membranes with 0.1% (v/v) Triton X-100 resulting in the completely unquenched probe (at 1,000 seconds).

CPP adsorption at the air-water interface

"Monolayer techniques" are potent tools for studying the interfacial properties of membrane-active peptides in which the membrane hydrophobic-hydrophilic interface could be mimicked by an air-water interface or a monolayer of phospholipids as described (Konate et al. 2010; Deshayes et al. 2011). With this technique we carry out adsorption tests which consist in measuring surface pressure (Π) variations induced by increasing amounts of peptide at the air-water interface. Surface pressure was obtained at equilibrium, t = 30 minutes, after injection of aliquots of an aqueous solution of peptide into the aqueous sub phase (0.154 M NaCl solution), gently stirred with a magnetic stirrer. The maximal concentration for which no further variation of surface pressure is detected corresponds to the critical micellar concentration (CMC) indicative of the air-water interface affinity and amphipathic features of peptides. Adsorptions at the air-water interface were performed with a homemade setup in which surface tension was measured with a Prolabo (France) tensiometer by using the platinum plate of the Wilhelmy method.

Tryptophan fluorescence spectrometry

Fluorescence experiments were performed on a PTI spectrofluorimeter at 25°C in 5% glucose. Intrinsic Tryptophan fluorescence of RICK at 5 µM was excited at 290 nm and emission spectrum was recorded between 320 and 390 nm, with a spectral band-pass of 2 and 6 nm for excitation and emission, respectively. LUVs of DOPC/SM/Chol (20:20:10, mol/mol/mol) were added to peptide solution at a final

lipid/peptide molar ratio of r = 80. All spectra are normalized to maximum of fluorescence of free peptides and plotted in relative fluorescence (%).

Atto633-labeling of the RICK peptide

For the Atto633 labeling, a RICK peptide bearing an N-terminal Cysteine-residue has been purchased from LifeTein. This peptide was reacted with a 2 molar excess of ATTO 633 maleimide (Atto Tec, Germany) during 5 h at room temperature. The Atto-peptide was then purified by RP-HPLC on a C18 column (Interchrom UP5 WOD/25 M Uptispere 300 5 ODB, 250 x 21.2 mm). The identity was assessed by Electrospray ionization mass spectra (Micromass Q-Tof, Waters).

Experiments on Giant Unilamellar Vesicles (GUVs)

GUVs were formed by using the hydration method (Weinberger et al, 2013). A 5% PolyVinylAlcohol, PVA (Sigma-Aldrich), solution is prepared (w/w) in ultra-pure water and homogeneously distributed over a coverslip (28 mm of diameter) which is left to dry for 30 min at 50°C. In the meantime, a lipids solution were prepared, composed in molar ratio of 1% of TopFluorPC (1-palmitoyl-2-(dipyrrometheneboron difluorideundecanoyl-sn-glycero-3-phosphocholine) and 99% of POPC (1-palmitoyl,2-oleoylphosphatidylcholine) beforehand dissolved in chloroform/methanol (3/1, v/v) mixture. We choose a simple lipid composition to prevent a phase separation during the formation of GUVs. The lipids solution is spread on the PVA film formed over coverslip and left for 2 min on a 40°C hotplate. Then the coverslip were placed for 2 h at room temperature into vacuum to evaporate all the solvent from lipids. The coverslip was placed in an attofluor chamber and a 280 mM sucrose solution (500 – 700 µl) was then added on the dried lipids and the coverslip chamber is left for 2 hours at 37°C to swell the lipids. The mixture was then placed overnight at room temperature until the GUV bilayers are formed. The obtained GUVs were transferred into an Eppendorf tube and diluted 9 times in a 5% glucose solution. For the microscopy

experiments, the GUVs are placed into a glass bottom cell culture dish (Greiner bio-one, dish diameter 35 mm, 4 compartments) and incubated with the nanoparticles (400 nM Atto633-RICK:20 nM siRNA).

Confocal images of GUVs were obtained with an inverted LSM780 multi-photon microscope (Zeiss). A HeNe excitation laser is used to illuminate a 40X (1.3 numerical aperture) oil immersion objective. Acquisition parameters for the different fluorophores are described below:

Fluorophore	Excitation (nm)	Emission band (nm)
TopFluorPC	488	500 – 524
Cy3b	561	560 – 595
Atto633	633	630 - 675

No emission bleed through was observed between the different channels observed with these acquisition parameters. Emission photons were collected, through a 600 µm pinhole, on PhotoMultiplierTube (PMT) which allows an accurate photons counting. The obtained confocal images were projected and treated with the software ImageJ.

Confocal microscopy on U87 living cells:

The following acquisition parameters were chosen to record fluorescent NPs behavior inside living cells (U87-LucF/LucR):

	Excitation laser (nm)	Wavelength of detection (nm)	Exposition time (ms)	Laser power (%)
Bright field images			300	
Cy3b	561	640	200	4
Atto633	590	695	10	1

Circular dichroism of CADY-K, D-cady-k and RICK mixtures

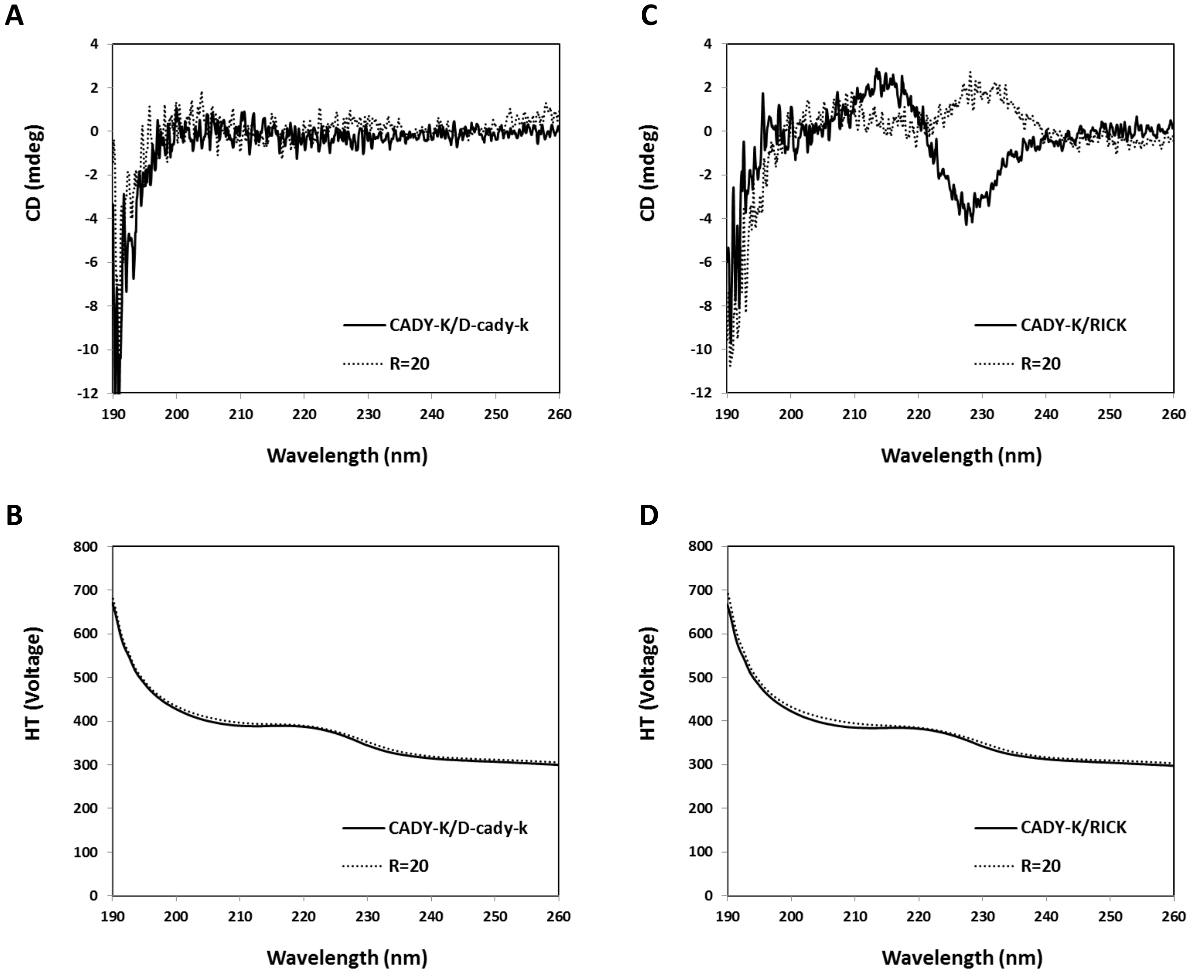

Figure S1: Circular dichroism spectra of CADY-K/D-cady-k (**A, B**) and CADY-K/RICK (**C, D**) mixtures. CADY-K is mixed with its D-isoform D-cady-k or *retro inverso* isoform RICK at an equimolar ratio and siRNA is then added on the mix to obtain a final peptide:siRNA ratio R = 20. CD signals are expressed in mdeg (**A** and **C**) and are associated to their corresponding high tension voltage (**B** and **D**).

Circular dichroism of folded and unfolded RICK vs D-cady-k

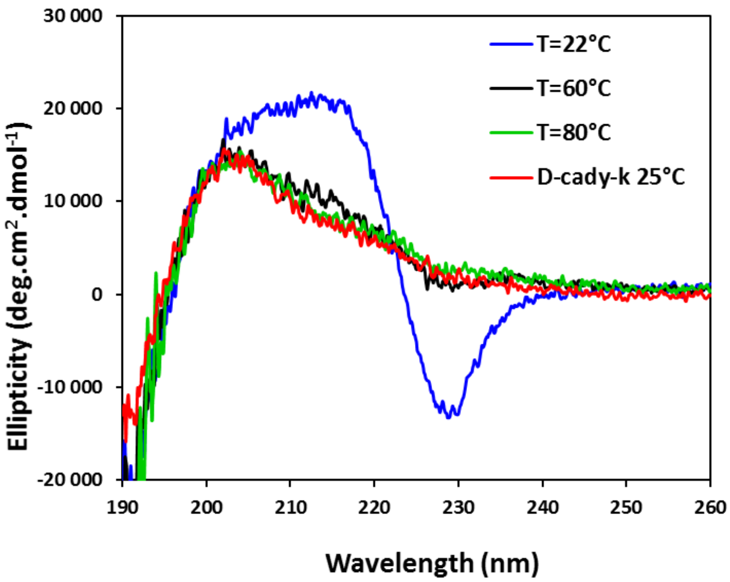

Figure S2: Superposition of the circular dichroism spectra of RICK at 22°C (blue), 60°C (black) and 80°C (green) and of D-cady-k at 25°C (red). All spectra were revealed at a peptide concentration of 4 µM. CD signals are expressed in Ellipticity.

DLS spectra of peptide-based nanoparticles

A

B

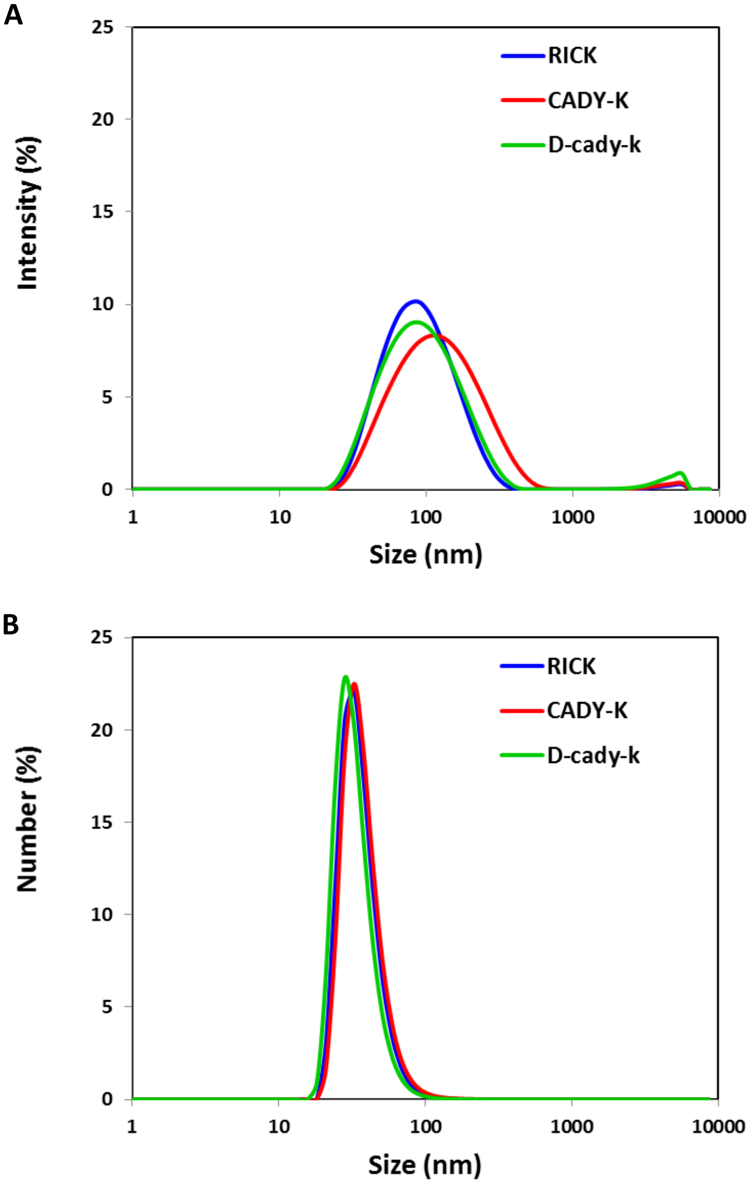

<u>**Figure S3:**</u> Examples of DLS size distribution of RICK:siRNA, CADY-K:siRNA and D-cady-k:siRNA (R = 20 with 500 nM siRNA) expressed in percentages of Intensity (A) or Number (B).

Transmission electronic microscopy (TEM)

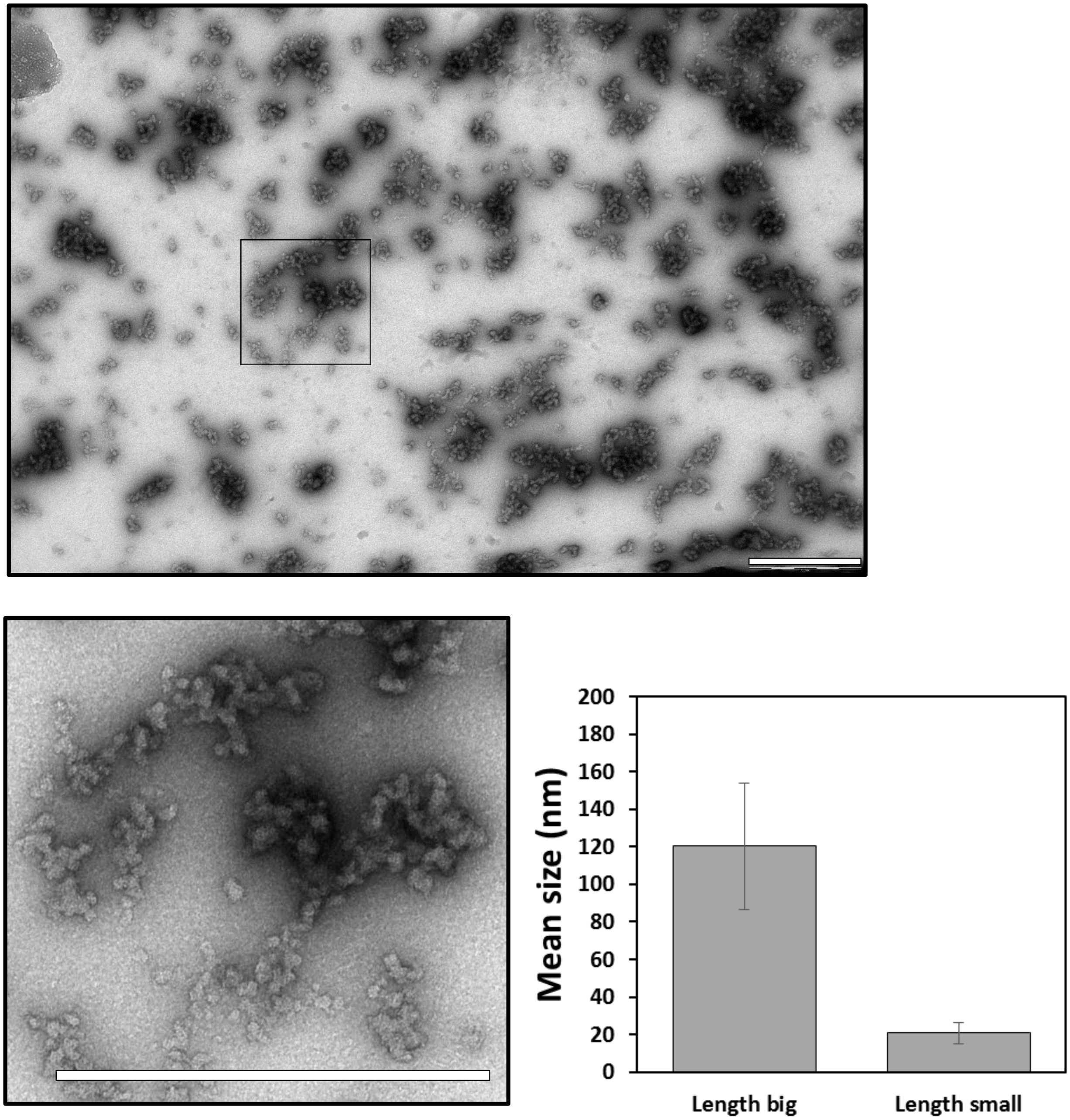

<u>Figure S4:</u> Example of transmission electron microscopy pictures obtained for RICK:siRNA nanoparticles. Size measurements using ImageJ indicated that big and small nanoparticles have a diameter of 120 ± 34 nm and 21 ± 6 nm, respectively. Scale bars correspond to 500 nm.

Mass spectra analyses

To confirm that the degradation products of CADY-K are due to the trypsin incubation, we collected the corresponding HPLC fractions for MS/MS analysis using a mass spectrometer (SYNAPT G2-S, Waters) equipped with an ESI source. Data acquisition was performed in positive ion mode in a mass range between 100 and 2000 Da. Interface parameters were set as follows: capillary voltage 3 kV and cone voltage 30 V. MS/MS spectra were recorded with cone voltage at 30 V and collision energy ramp between 15 eV and 60 eV was used. The data confirmed the expected digestion products of CADY-K.

CADY-K sequence: GLWR / ALWR / LLR / SLWR / LLWK

<u>Table S1</u>: MS/MS analysis of the collected HPLC fractions issue from CADY-K digestion.

Expected CADY-K digestion products	Molecular weight (Da)		MS/MS ions (Da)	
	Calculated MH^+ (mono)	found	calculated	found
GLWR	531.3038	531.30	**y1** 175.1190	175.12
			**y2** 361.1983	361,20
			**y3-NH3** 457.2558	457,25
ALWR	545.3194	545.31	**y1** 175.1190	175.12
			**y2** 361.1983	361.20
			**y3** 474.2823	474.29
LLR	401.2871	-	-	-
SLWR	561.3144	561.32	**y1** 175.1190	175.12
			**y2** 361.1983	361.20
			**a2** 173.1285	173.13
			**y3** 474.2823	457.26
LLWK	559.3602	559.37	**b3** 413.2547	413.26
			**y2** 333.1921	333.19
			**y3** 446.2762	446.27

Characterization of the membrane interaction of RICK alone or complexed to siRNA

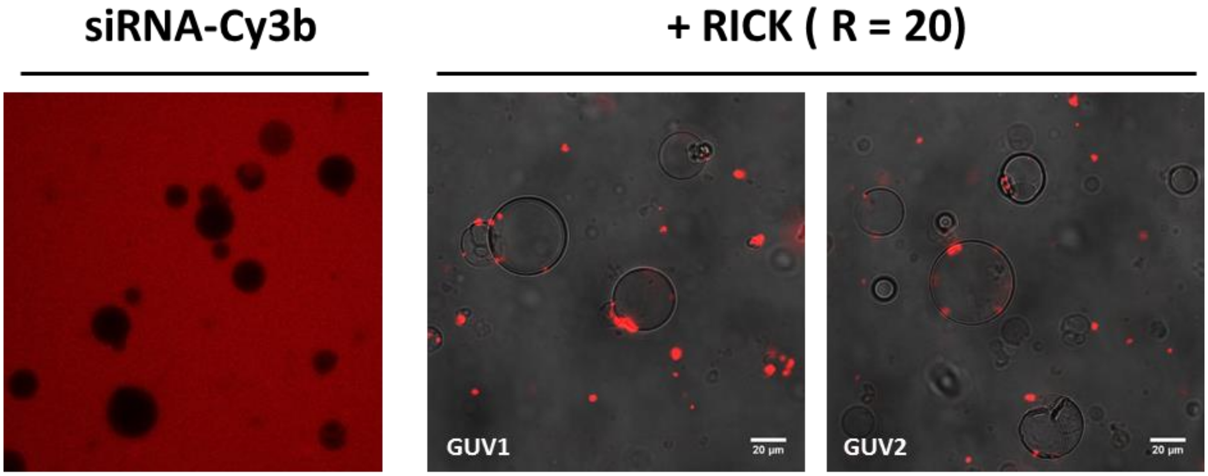

Figure S5: Membrane interaction of successively formulated RICK:siRNA nanoparticles on GUVs. Representative fluorescence microscopy images of GUVs incubated with 20 nM siRNA-Cy3b alone (left hand panel). Thereafter, 400 nM Atto633-RICK was added to the siRNA/GUV solution. After 80 min incubation, we observe a dotted pattern of siRNA-Cy3 which corresponds to the RICK nanoparticles as shown on two different images (GUV1 and GUV2). Bars represent 20 µm.

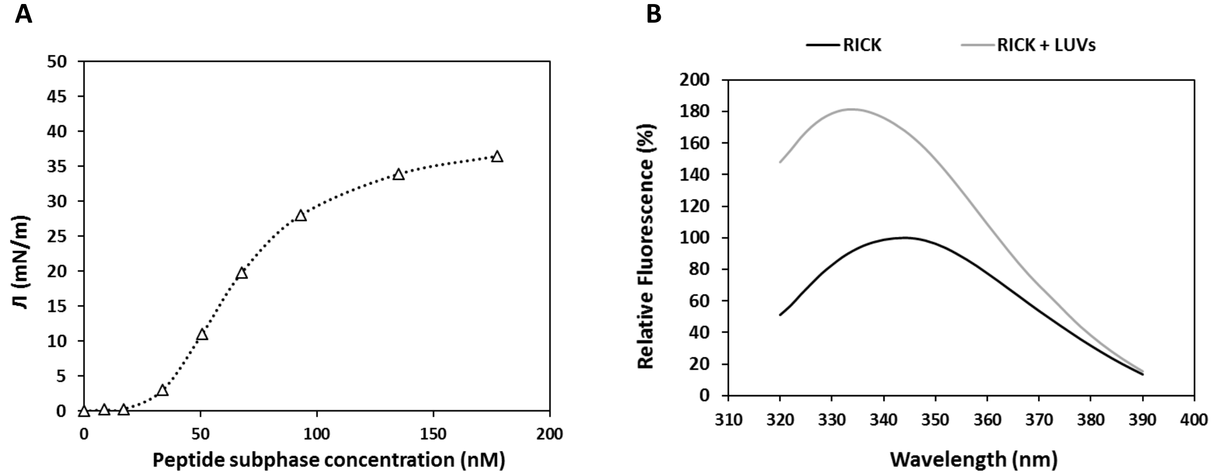

<u>Figure S6</u>: (**A**) Adsorption curves of RICK at the air-water interface (**B**) Intrinsic tryptophan fluorescence spectra of free RICK peptide and for RICK in the presence of LUVs at a lipid:peptide molar ratio of r = 80 in 5% glucose.

Principle of the Dual luciferase evaluation

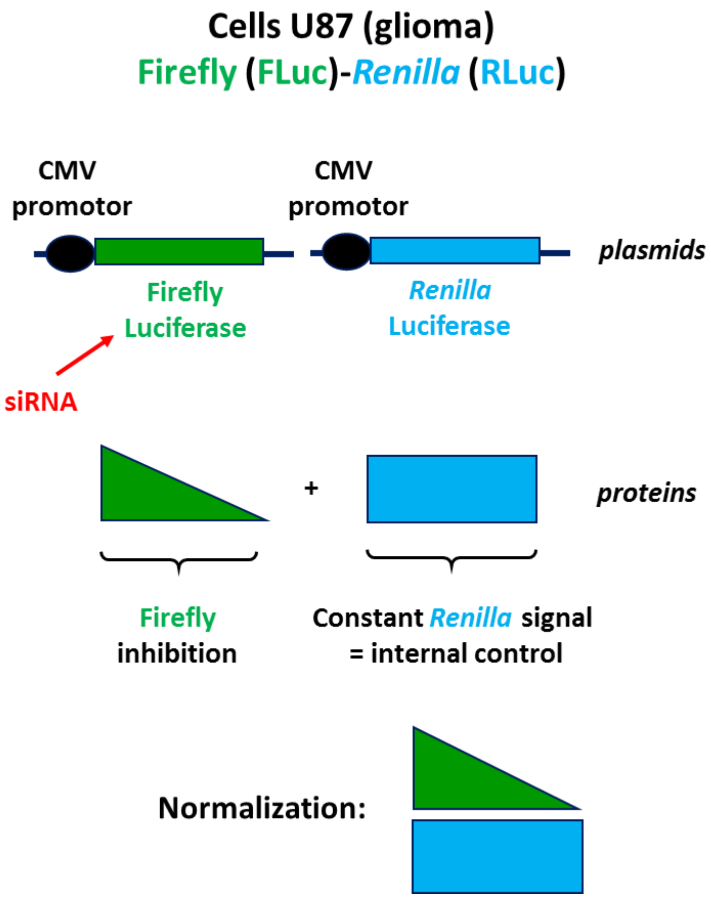

<u>Figure S7</u>: Scheme of the plasmid used to stably transfect the U87 cell line.
